# Ascorbate-glutathione cycle in wheat and rice seedlings under anoxia and subsequent reaeration

**DOI:** 10.18699/vjgb-24-06

**Published:** 2024-02

**Authors:** V.V. Yemelyanov, E.G. Prikaziuk, V.V. Lastochkin, O.M. Aresheva, T.V. Chirkova

**Affiliations:** Department of Genetics and Biotechnology, Faculty of Biology, Saint Petersburg State University, St. Petersburg, Russia Department of Plant Physiology and Biochemistry, Faculty of Biology, Saint Petersburg State University, St. Petersburg, Russia; Department of Plant Physiology and Biochemistry, Faculty of Biology, Saint Petersburg State University, St. Petersburg, Russia Department of Water Resources, ITC Faculty of Geo-Information Science and Earth Observation, University of Twente, Enschede, the Netherlands; Department of Plant Physiology and Biochemistry, Faculty of Biology, Saint Petersburg State University, St. Petersburg, Russia; Department of Plant Physiology and Biochemistry, Faculty of Biology, Saint Petersburg State University, St. Petersburg, Russia; Department of Plant Physiology and Biochemistry, Faculty of Biology, Saint Petersburg State University, St. Petersburg, Russia

**Keywords:** anoxia, reaeration, oxidative stress, ascorbate, glutathione, ascorbate-glutathione cycle, wheat, rice, аноксия, реаэрация, окислительный стресс, аскорбиновая кислота, глутатион, аскорбат-глутатионовый цикл, пшеница, рис

## Abstract

The most important part of the plant antioxidant system is the ascorbate-glutathione cycle (AGC), the activity of which is observed upon exposure to a range of stressors, including lack of O2, and oxidative stress occurring immediately after the restoration of oxygen access, hereafter termed reaeration or post-anoxia. The operation of the AGC (enzymes and low-molecular components) in wheat (Triticum aestivum, cv. Leningradka, non-resistant to hypoxia) and rice (Oryza sativa, cv. Liman, resistant) seedlings after 24 h anoxia and 1 h or 24 h reaeration was studied. Significant accumulation of oxidized forms of ascorbate and glutathione was revealed in the non-resistant plant (wheat) after 24 h of anoxia and reaeration, indicating the development of oxidative stress. In the resistant plant (rice), reduced forms of these antioxidants prevailed both in normoxia and under stress, which may indicate their intensive reduction. In wheat, the activities of ascorbate peroxidase and dehydroascorbate reductase in shoots, and monodehydroascorbate reductase and glutathione reductase in roots decreased under anoxia and reaeration. The activity of antioxidant enzymes was maintained in rice under lack of oxygen (ascorbate peroxidase, glutathione reductase) and increased during post-anoxia (AGC reductases). Anoxia stimulated accumulation of mRNA of the organellar ascorbate peroxidase genes OsAPX3, OsAPX5 in shoots, and OsAPX3-5 and OsAPX7 in roots. At post-anoxia, the contribution of the OsAPX1 and OsAPX2 genes encoding the cytosolic forms of the enzyme increased in the whole plant, and so did that of the OsAPX8 gene for the plastid form of the enzyme. The accumulation of mRNA of the genes OsMDAR2 and OsMDAR4 encoding peroxisomal and cytosolic monodehydroascorbate reductase as well as the OsGR2 and OsGR3 for cytosolic and organellar glutathione reductase was activated during reaeration in shoots and roots. In most cases, O2 deficiency activated the genes encoding the peroxisomal, plastid, and mitochondrial forms of the enzymes, and upon reaeration, an enhanced activity of the genes encoding the cytoplasmic forms was observed. Taken together, the inactivation of AGC enzymes was revealed in wheat seedlings during anoxia and subsequent reaeration, which disrupted the effective operation of the cycle and triggered the accumulation of oxidized forms of ascorbate and glutathione. In rice, anoxia led to the maintenance of the activity of AGC enzymes, and reaeration stimulated it, including at the level of gene expression, which ensured the effective operation of AGC

## Abstract (ru)

Важной частью антиоксидантной системы растений является аскорбат-глутатионовый цикл (АГЦ), функционирование которого регистрируется при действии разнообразных стрессоров, в том числе дефицита и/ или отсутствия O2, а также окислительного стресса, возникающего сразу после восстановления доступа кислорода (реаэрация, или постаноксия). Показана значительная аккумуляция окисленных форм аскорбата и глутатиона в проростках пшеницы (Triticum aestivum, сорт Ленинградка, неустойчивое растение) при действии на него 24-часовой аноксии и реаэрации, что свидетельствует о развитии окислительного стресса. У риса (Oryza sativa, сорт Лиман, устойчивое растение) преобладали восстановленные формы данных антиоксидантов как в контроле, так и при стрессе, что может указывать на их интенсивное восстановление. У пшеницы активности аскорбатпероксидазы и дегидроаскорбатредуктазы в побегах, а также монодегидроаскорбатредуктазы и глутатионредуктазы в корнях снижались под действием аноксии и реаэрации. У риса активность антиоксидантных ферментов сохранялась в отсутствие кислорода (аскорбатпероксидаза, глутатионредуктаза) и возрастала при постаноксии (редуктазы АГЦ). Аноксия стимулировала накопление мРНК генов органелльных форм аскорбатпероксидазы OsAPX3, OsAPX5 в побегах и OsAPX3-5, OsAPX7 в корнях проростков риса. При постаноксии во всем растении возрастал вклад генов цитоплазматических форм фермента – OsAPX1, OsAPX2, а также пластидной OsAPX8. При реаэрации аккумулировались транскрипты пероксисомной и цитоплазматической монодегидроаскорбатредуктазы OsMDAR2 и OsMDAR4, цитозольной и органелльной глутатионредуктазы OsGR2 и OsGR3. В большинстве случаев недостаток О2 индуцировал активность генов, кодирующих пероксисомные, пластидные и митохондриальные формы ферментов, а реаэрация усиливала работу генов, кодирующих цитоплазматические формы. При действии аноксии и последующей реаэрации выявлена инактивация ферментов АГЦ в проростках пшеницы, что нарушало эффективную работу цикла и запускало аккумуляцию окисленных форм аскорбата и глутатиона. У риса аноксия приводила к сохранению активности ферментов АГЦ, а реаэрация ее стимулировала, в том числе на уровне экспрессии генов, что обеспечивало эффективное функционирование аскорбат-глутатионового цикла.

## Introduction

Plants have a multi-level system of protection against the damaging effects of reactive oxygen species (ROS), which accumulated in response to changes in environmental conditions, developmental stages, the action of hormones, etc. (Halliwell, 2006; Foyer, Noctor, 2009; Shikov et al., 2021). An important part of the antioxidant system is the ascorbateglutathione cycle (AGC, Foyer–Halliwell–Asada pathway), which ensures the effective reduction of low-molecular-weight antioxidants – ascorbate and glutathione. Ascorbic acid (AsA, vitamin C) is a key water-soluble antioxidant present in all cell compartments, including the apoplast (Noctor, Foyer, 1998). AsA neutralizes most ROS and reduces tocopherols and epoxycarotenoids of the violaxanthin cycle. When AsA is oxidized, either a short-lived radical, monodehydroascorbic acid (MDA), or a non-radical oxidized form, dehydroascorbic acid (DHA), is formed. Two molecules of MDA can undergo a disproportionation reaction to form one molecule of reduced AsA and one molecule of DHA.

Glutathione, another low-molecular-weight AGC antioxidant, is a γ-Glu-Cys-Gly tripeptide. It is present throughout the cell, but in the apoplast, only in trace amounts (Noctor, Foyer, 1998; Gill, Tuteja, 2010). Glutathione neutralizes reactive oxygen and nitrogen species, free radicals and fatty acid peroxides, participates in the neutralization of methylglyoxal, xenobiotics and heavy metals, and reduces DHA and sulfhydryl groups (Hasanuzzaman et al., 2017). It is involved in various physiological processes, including redox regulation, signal transduction, conjugation and transport of metabolites, regulation of plant growth and development (Gill, Tuteja, 2010).

The key enzyme of the AGC, ascorbate peroxidase (APX, EC 1.11.1.11), oxidizes two AsA molecules to two MDA. APX belongs to class I haem peroxidases and is localized mainly in plastids (most isoforms), as well as in the cytoplasm and peroxisomes. A number of stromal APXs were also found in the mitochondrial matrix (Ishikawa, Shigeoka, 2008). MDA in the AGC can be reduced to AsA by monodehydroascorbate reductase (MDAR, EC 1.6.5.4), localized in the cytosol, peroxisomes, plastids and mitochondria. When reducing two molecules of MDA, MDAR oxidizes one molecule of NADH or NADPH. DHA can be reduced non-enzymatically by glutathione, especially at the alkaline pH values found in the chloroplast stroma. Thioredoxins f and m also reduce DHA (Morell et al., 1997). In the AGC, dehydroascorbate reductase (DHAR, EC 1.8.5.1) is responsible for the reduction of DHA, while two molecules of reduced glutathione (GSH) are converted into an oxidized form – glutathione disulfide (GSSG). DHAR belongs to the glutathione S-transferase superfamily, although it is not capable of catalyzing glutathione conjugation or peroxide reduction. DHAR is localized in the cytosol, peroxisomes, plastids and mitochondria, and in a number of plants – in the apoplast and vacuoles (Ding et al., 2020). Glutathione reductase (GR, EC 1.8.1.7) catalyzes the reduction of glutathione at the expense of NADPH. GR functions in the cytoplasm, plastids and mitochondria, with 80 % of the activity registered in photosynthetic tissues occurring in chloroplasts (Gill et al., 2013). Data from proteomic analysis confirm the presence of GR in peroxisomes (Palma et al., 2009). GR is not only involved in the AGC, but also in the maintenance of sulfhydryl groups and the reduction of glutathione, oxidized directly by ROS, fatty acid peroxides and S-conjugates, etc. (Gill, Tuteja, 2010; Gill et al., 2013).

The AGC was formulated in relation to the antioxidant protection of the photosynthetic apparatus (Foyer, Halliwell, 1976). However, the cycle also operates in the cytosol and, partially, in peroxisomes and mitochondria. The AGC is important for plant adaptation to adverse environmental factors. Increased activity and gene expression of most AGC enzymes is characteristics for plants that are resistant to drought, salinity, non-optimal temperatures, heavy metals, phytopatho- gens, etc. Transgenic plants overexpressing genes of the AGC show increased resistance to stressors (Hasanuzzaman et al., 2019).

One of the common stress factors affecting plants is the deficiency (hypoxia) or complete absence (anoxia) of oxygen, occurring in wet or waterlogged soils, during inundation or flooding (Chirkova, Yemelyanov, 2018). ROS are formed in an oxygen-free environment, where they participate in the transduction of an anaerobic signal and trigger destructive processes (Blokhina et al., 2001). Subsequent oxidation of reduced products accumulated during anoxia leads to increased generation of ROS and the development of post-ano-xic oxidative damage (Blokhina et al., 2003; Shikov et al., 2020).

There are numerous data on the changes in the operation of AGC components under the influence of hypo- and anoxia. O2 deficiency led to a decrease in the activity of all AGC enzymes in hypoxically grown rice and lotus seedlings (Ushimaru et al., 1992, 2001), and in wheat roots (Biemelt et al., 1998). However, no changes in enzymatic activity were detected in the roots of lupine (Lupinus luteus) (Garnczarska, 2005). Prolonged waterlogging even stimulated most AGC enzymes in citrumelo (Citrus paradisi L. Macf. × Poncirus trifoliata L. Raf.) (Hossain et al., 2009). A decrease in AGC enzymes activity under flooding was observed in soybean roots – APX (Kausar et al., 2012), in Welsh onion – APX and GR (Yiu et al., 2009), and in cotton leaves – APX, DHAR and GR (Wang et al., 2019). Reaeration, on the contrary, stimulated most AGC enzymes (Ushimaru et al., 1992, 2001; Garnczarska, 2005).

Only a handful of studies compared the operation of the AGC between plants with different levels of resistance to oxygen deficiency. In leguminous plants, pigeon pea (Cajanus cajan) and mung bean (Vigna radiata), varieties resistant to flooding were characterized by increased activity of APX and GR under hypoxia and reaeration, which was accompanied by upregulated expression of the corresponding genes (Sairam et al., 2009, 2011). An increase in APX activity during submergence was reported for tolerant slow-growing varieties of rice (Damanik et al., 2010) and ryegrass (Lolium perenne) (Liu, Jiang, 2015). Anoxia and reoxygenation caused greater ROS production and oxidative damage to lipids and proteins in seedlings of a non-resistant wheat plant (Chirkova et al., 1998; Shikov et al., 2022), and an increased activity of catalase and class III peroxidase in a resistant plant (rice) (Yemelyanov et al., 2022).

The objective of this study was to analyze the effects of anoxia and post-anoxic oxidative stress on the content of lowmolecular- weight antioxidants and the activity of enzymes of the ascorbate-glutathione cycle in wheat and rice plants.

## Materials and methods

Plant material. The objects of the study were 7-day-old wheat seedlings (Triticum aestivum L.) of the Leningradka variety and 10-day-old rice seedlings (Oryza sativa L.) of the Liman variety. Wheat caryopses were purchased from the Suida Breeding Station (Leningrad Region, Russia), rice seeds were provided by the Federal Rice Research Center (Krasnodar, Russia). Wheat was used as a plant that is not resistant to hypoxia, and rice was used as a hypoxia-tolerant one.

The seeds were surface-sterilized with a 5 % sodium hypochlorite solution, germinated, and seedlings were grown in hydroponic culture, as we previously described (Yemelyanov et al., 2020, 2022). Anaerobic conditions were created by passing nitrogen gas (oxygen content <0.01 %, Lentekhgaz, Russia) through chambers with plants, which were then hermetically sealed and placed in the dark to prevent the formation of oxygen in the light. Anaerobic conditions were checked using the Anaerotest® indicator (Merck, Germany). Exposure to a nitrogen atmosphere was 24 hours. Control plants were placed in the dark under normoxic conditions. Next, to create post-anoxia, the experimental plants were removed from the anaerobic chambers and transferred into ambient air in the dark for 15 minutes, 1, 3 and 24 hours in experiments to determine low-molecular antioxidants, and 1 and 24 hours in the experiments to study the activity of enzymes of the ascorbate-glutathione cycle and expression of corresponding genes

Concentrations of ascorbic acids and glutathione. Lowmolecular- weight antioxidants of the AGC were extracted with chilled 5 % metaphosphoric acid from 1 g of shoots and roots after homogenization in liquid nitrogen (Blokhina et al., 2000). The extract was filtered and centrifuged for 30 minutes at 15,000 g. The content of ascorbate and dehydroascorbate was determined spectrophotometrically using the bipyridyl method (Knörzer et al., 1996). Reduced and oxidized glutathione was detected enzymatically (Law et al., 1983; Knörzer et al., 1996) using glutathione reductase (Sigma, USA).

Activities of AGC enzymes. To extract enzymes, shoots and roots of 10 seedlings were weighed and ground with a mortar and pestle in a chilled buffer (tissue : buffer ratio was 1:10, w/v) with the addition of quartz sand. All operations were performed at +4 °C. Determination of the protein content in the enzyme extract was carried out using the Bradford method (Bradford, 1976). When calculating enzyme activity, the autoxidation of substrates was taken into account, which was recorded without the addition of enzyme extract.

To extract ascorbate peroxidase, plant tissue was homogenized and extracted with 0.05 M K,Na-phosphate buffer (pH 7.0). After 15 minutes of centrifugation at 8,000 g, the supernatant was collected, and the pellet was resuspended with 1/2 of the original volume of buffer, then extracted for 20 minutes and centrifuged again. The combined fraction was used to determine enzyme activity by the decrease in absorption at 290 nm (spectrophotometer SP-26, LOMO, Russia) due to ascorbate oxidation (Nakano, Asada, 1981). The reaction medium for determining APX activity consisted of 40 μl of enzyme extract (7.0–23.2 μg of protein) in 0.05 M K,Na-phosphate buffer (pH 7.0), to which 0.5 ml of 5 mM ascorbate (Sigma) was added. The reaction was initiated by adding H2O2 (2.5 mM); distilled water was added to the control variant. The final volume of the reaction medium was 3 ml. Enzyme activity was calculated in micromoles of ascorbate oxidizable per 1 g of fresh weight per min using the extinction coefficient of 2.8 mM–1 · cm–1.

Intermediate AGC reductases (MDAR and DHAR) were extracted with 0.05 M K,Na-phosphate buffer (pH 7.3) containing 2 mM EDTA and 1 % cross-linked polyvinylpolypyrrolidone (Sigma). The homogenate was filtered and centrifuged at 15,000 g for 20 minutes.

MDAR activity was determined in a reaction coupled with ascorbate oxidase (Arrigoni et al., 1981). The composition of the reaction medium was 100 μl of 0.3 mM ascorbic acid, 200 μl of 0.3 mM NADH (Sigma), 0.5 units of ascorbate oxidase (Sigma), 300 μl of the sample (shoot extract) or 500 μl (root extract) and 0.05 M K,Na-phosphate buffer (pH 6.3). The final volume of the medium was 3 ml. The reaction was initiated by adding ascorbate oxidase. Optical density was measured at 340 nm with an SP-46 spectrophotometer (LOMO, Russia). Enzyme activity was calculated in nanomoles of oxidizable NADH per 1 g of fresh weight per minute (NAD(P) H extinction coefficient was 6.22 mM–1 · cm–1).

DHAR activity was detected in the reaction medium containing 100 μl of 0.5 mM dehydroascorbic acid (Sigma), 100 μl of 1 mM reduced glutathione (Sigma), 50 μl of enzyme extract (from the shoots) or 100 μl (from the roots) and 0.05 M K,Na-phosphate buffer (pH 7.0) (Knörzer et al., 1996). The final volume was 3 ml. The reaction was initiated by adding DHA, which was dissolved in distilled water saturated with nitrogen gas immediately prior to measuring the activity. Optical density was measured at 265 nm with an SP-46 spectrophotometer. Enzyme activity was calculated in micromoles of reduced ascorbate per 1 g of fresh weight per minute.

Glutathione reductase was extracted with 0.1 M K,Naphosphate buffer (pH 7.5) containing 2 mM EDTA and 1 % cross-linked polyvinylpolypyrrolidone. The homogenate was filtered and centrifuged at 15,000 g for 20 minutes. GR activity was determined in the following medium: 300 μl of extract, 100 μl of 0.5 mM oxidized glutathione (Sigma), 200 μl of 0.2 mM NADPH (Sigma) in 0.1 M K,Na-phosphate buffer (Rao et al., 1995). The final volume of the medium was 3 ml. The reaction was initiated by adding NADPH. Optical density was measured at 340 nm with an SP-46 spectrophotometer. Enzyme activity was calculated in nanomoles of NADPH oxidizable per 1 g of fresh weight per minute

Gene expression was studied in rice seedlings, since the activity of most of the studied enzymes maintained or increased under stress. To design primers, we used the annotated rice genome databases (Rice Genome Annotation Project, http:// rice.uga.edu/, last accessed 22 December 2023, and The rice annotation project database, http://rapdb.dna.affrc.go.jp/tools/ search/, last accessed 22 December 2023), as well as the rice reference genome IRGSP-1.0 Oryza sativa var. japonica cv. Nipponbare, available at http://www.ncbi.nlm.nih.gov/ datasets/genome/GCF_001433935.1/ (last accessed 22 December 2023).

Nucleotide sequences of genes encoding all types of ascorbate peroxidases (8 genes), monodehydroascorbate reductases (5 genes), dehydroascorbate reductases (2 genes) and glutathione reductases (3 genes) were found. Coding DNA sequences (CDS) were examined. Among ascorbate peroxidases, several CDS were identified for the OsAPX8 gene (2); among monodehydroascorbate reductases – for the OsMDAR2 and OsMDAR4 genes (2 each); among glutathione reductases – for OsGR2 (3) and OsGR3 (2). All CDS of the corresponding genes were aligned using the ClustalW algorithm in the MegAlign 5.05 program from the DNAStar suite. Primers were designed for the consensus regions closest to the 3ʹ end of the CDS in the VectorNTI 8 program (Supplementary Materials 1 and 2)1, i. e. the primers we developed allow to evaluate the expression of any alternatively spliced variants of the genes of interest. OsTUB4, encoding β-tubulin-4 and demonstrating the most stable expression, was used as a reference gene (Yemelyanov et al., 2022). The specificity of the primers was checked by searching for homology with the rice genome and transcriptome using the BLASTn algorithm on the NCBI database website (http://blast.ncbi.nlm.nih.gov/, last accessed 22 December 2023). Primers were ordered from the Beagle company (Russia, http://www.biobeagle.com/, last accessed 22 December 2023)


Supplementary Materials are available in the online version of the paper: https://vavilov.elpub.ru/jour/manager/files/Suppl_Emel_Engl_28_1.pdf


Methods for RNA isolation and purification, reverse transcription and quantitative real-time PCR (RT-PCR) have been described in details previously (Yemelyanov et al., 2022). RT-PCR was performed using kits with SYBRGreen dye (Syntol, Russia, http://www.syntol.ru, last accessed 22 December 2023) in a C1000 thermal cycler with a CFX96 optical module (Bio-Rad Laboratories, USA) according to the manufacturer’s recommendations. We used the equipment of the Center for Molecular and Cell Technologies of Research park of St. Petersburg State University

The 2–ΔCt method was used to obtain the relative amount of transcripts from the difference in threshold amplification cycles (Ct) between the target and the reference gene (OsTUB4), and the 2–ΔΔCt method was used to obtain the degree of change in the relative amount of transcripts of each gene (Livak, Schmittgen, 2001). Changes in the expression level were calculated relative to control (normoxic) values, taking them as one

Statistical analysis. All experiments were carried out in 4–8 biological and 3 analytical replicates. Statistical data processing was performed using GraphPad Prism 8.0.1 for Windows. The graphs in figures show the average values and their standard errors. Values with different letters were significantly different at p < 0.05 (Tukey weighted mean). Heatmaps were generated using the tidyverse package (Wickham et al., 2019) in the R software environment (R Core Team, 2023). Asterisks on the heatmap indicate statistically significant differences from the control (Mann–Whitney U test, p < 0.05).

## Results

The impact of anoxia and reoxygenation on the low-molecular-weight antioxidants of the AGC

**Fig. 1.**
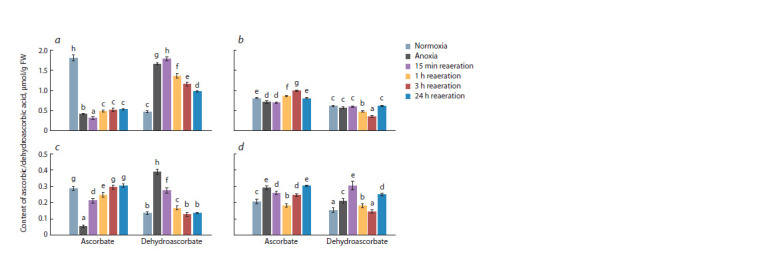
Effect of 24 h anoxia and subsequent reaeration on the ascorbic acid (AsA) and dehydroascorbic acid (DHA) content in shoots (a, b) and roots (c, d ) of wheat (a, c) and rice (b, d ) seedlings. Values with different letters (a–h) are significantly different at p < 0.05, according to Tukey’s test.

The initial level of ascorbate in wheat was higher than in rice (twice as high in the shoots and 1.5 times in the roots, Fig. 1). The reduced form of ascorbate (AsA) dominated in the seedlings of both plants (80 and 70 % in wheat shoots and roots, respectively, and 60 % in the organs of the rice seedlings). The 24-hour anoxia caused a 4-fold decrease in the level of AsA in the shoots and a 5-fold decrease in the roots of the wheat seedlings (see Fig. 1, a, c), accompanied by the accumulation of dehydroascorbate (DHA) (increasing by 3.5 and 3 times, respectively). As a result, the oxidized form dominated, accounting for 80 % in the shoots and 90 % in the roots of wheat seedlings. In the shoots of rice, under the influence of anoxia, the content of AsA decreased by 10 %, and DHA remained unchanged (see Fig. 1, b). Meanwhile, in the roots, the level of both ascorbate forms increased by 40 % (see Fig. 1, d ). A 15-minute reoxygenation led to a further depletion in the AsA content and the accumulation of DHA in wheat shoots. A more prolonged post-anoxic period (1–24 hours) led to the accumulation of AsA and a decrease in DHA levels (see Fig. 1, c), with both forms reaching control values. Notably, the total level of ascorbate (AsA + DHA) decreased from 2.3 to 1.5 μmol/g fresh weight in wheat shoots under the influence of anoxia and subsequent reoxygenation, and it was preserved in the roots. Changes of ascorbate in rice seedlings were in the opposite direction: in the shoots, the level of AsA/ DHA initially increased/decreased (respectively) and then returned to the control level (see Fig. 1, b), while in the roots, conversely, the levels initially decreased/increased, but by the end of the experiment, both parameters exceeded control values (see Fig. 1, d ). The total level of ascorbate in rice seedlings did not change, except for the 24-hour reoxygenation, when it increased 1.5 times in the roots.

**Fig. 2.**
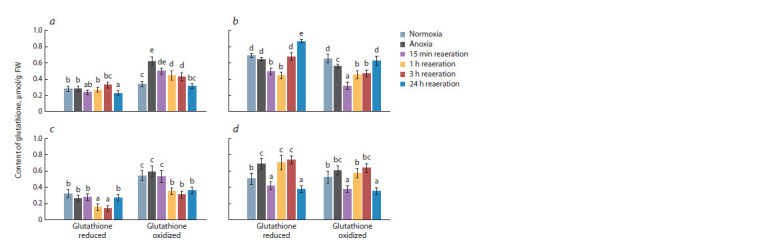
Effect of 24 h anoxia and subsequent reaeration on the reduced (GSH) and oxidized (GSSG) glutathione content in shoots (a, b) and roots (c, d) of wheat (a, c) and rice (b, d) seedlings. Values with different letters (a–e) are significantly different at p < 0.05, according to Tukey’s test.

Next, let us examine the dynamics of glutathione content. The initial concentrations of reduced glutathione (GSH) and total glutathione (GSH + GSSG) were higher in rice seedlings, particularly in the shoots (Fig. 2). The content of both forms, GSH and GSSG was approximately the same. The ratio of GSH/GSSG was 1.3 in the shoots and 1.1 in the roots of rice, while in wheat, the oxidized form (GSSG) dominated, with a GSH/GSSG ratio of 0.6 and 0.9, respectively. The lack of oxygen did not affect the GSH level in wheat seedlings and rice shoots, whereas in rice roots, it increased by 25 % (see Fig. 2, d ). The content of GSSG changed under anoxia only in the shoots of the studied plants: it increased twofold in wheat, while it decreased by 15 % in rice (see Fig. 2, а, b). The level of GSH decreased in wheat shoots after 24 hours of reoxygenation, and in the roots at shorter intervals (1–3 hours), after which it returned to the control level (see Fig. 2, а, c). During post-anoxia, the GSSG content in the shoots returned to the control level (normoxia) and was lower than under normoxic conditions in the roots. The level of total glutathione (GSH + GSSG) did not change significantly under stress, and the proportion of GSH decreased from 46 % (shoots) and 40 % (roots) under normoxia to 31–35 % under anoxia and short-term reoxygenation, after which it returned to control values. The post-anoxic period had a similar effect on the levels of both forms of glutathione in rice shoots. In the shoots, it decreased during short-term reoxygenation (15 minutes to 3 hours) and increased by 24 hours of post-anoxia (see Fig. 2, b). In rice roots, the content of both forms decreased after 15 minutes of reoxygenation, increased by 1–3 hours, and then decreased again by 24 hours of post-anoxia (see Fig. 2, d ). The total glutathione level in rice decreased during anoxia and short-term reoxygenation (15 minutes), and then returned to the control normoxic level. The GSH/GSSG ratio in rice shoots was greater than one throughou all the experiment, indicating a predominance of GSH.

The impact of anoxia and reoxygenation on the enzymes of the AGC

**Fig. 3.**
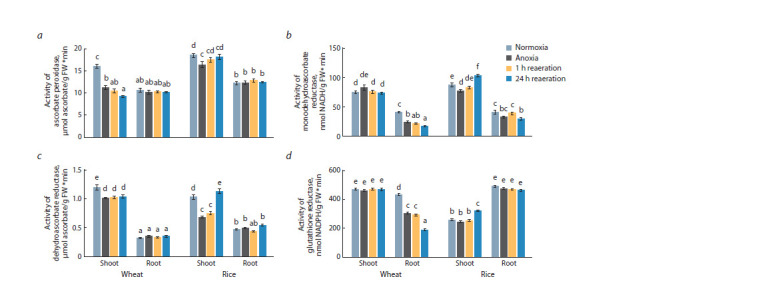
Activity of enzymes of the ascorbate-glutathione cycle in the shoots and roots of wheat and rice seedlings under 24 h anoxia and subsequent reaeration: a, ascorbate peroxidase (APX); b, monodehydroascorbate reductase (MDAR); c, dehydroascorbate reductase (DHAR); d, glutathione reductase (GR) Values with different letters (a–f ) are significantly different at p < 0.05, according to Tukey’s test.

Figure 3 depicts changes in the activity of high-molecularweight components of the AGC. Interestingly, the baseline activity level of AGC enzymes, except for rice GR, was higher in the shoots of plants of both species (see Fig. 3). Anoxia and subsequent reoxygenation led to a significant downregulation of APX activity in wheat shoots, while in rice shoots, a 10 % decrease in activity under oxygen deficiency was followed by a return to the control level during post-anoxia (see Fig. 3, a). In the roots of both species, neither anoxia nor reoxygenation caused changes in APX activity, similar to MDAR in wheat shoots (see Fig. 3, b). In wheat roots, the activity of MDAR decreased almost twofold as a result of oxygen deficiency and reoxygenation. In rice shoots, the change in MDAR activity was similar to that of APX, only after 24 hours of post-anoxia, the activity exceeded the control normoxic value. In rice roots, MDAR was consistently active throughout the entire experiment and was inhibited by 25 % only after 24 hours of reoxygenation (see Fig. 3, b).

The inactivation of DHAR was registered during anoxia in the shoots of both plants. However, in the case of rice during reoxygenation, the activity of the enzymes was restored and increased; however, this was not the case for wheat (see Fig. 3, c). In the roots of both plants, the activity of DHAR remained unchanged under all experimental conditions, similar to the activity of GR in wheat shoots and rice roots (see Fig. 3, d ). In wheat roots, the enzyme was deactivated under stress conditions, while in rice, it was stimulated after 24 hours of reoxygenation (see Fig. 3, d ).

The impact of anoxia and reoxygenation on the expression of genes encoding AGC enzymes in rice

**Fig. 4.**
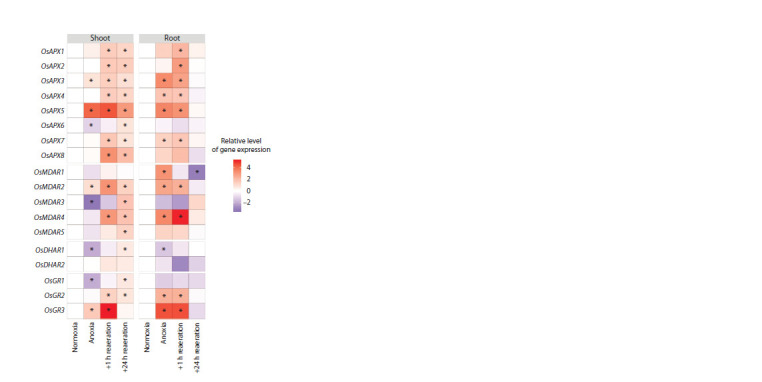
Heatmaps of relative transcript levels of genes encoding enzymes of ascorbate-glutathione cycle in the shoots and roots of rice seedlings under 24 hours of anoxia and subsequent reaeration. The expression level in the control (normoxia) is taken as one. Asterisks on the heatmap indicate statistically significant differences from the control (Mann– Whitney U test, p < 0.05).

An analysis of the expression of genes encoding AGC enzymes was conducted in rice tissues, where the activation of enzymes during post-anoxia was demonstrated. APX in rice is encoded by eight genes: OsAPX1 and 2 encode cytosolic isoforms, OsAPX3 and 4 – peroxisomal ones, OsAPX5 and 6 – isoforms with dual plastid-mitochondrial localization, OsAPX7 – the stromal isoform of plastids, OsAPX8 – the thylakoid one (see Supplementary Material 1). In rice shoots, 24-hour anoxia resulted in an insignificant increase in the expression of peroxisomal OsAPX3, a 15-fold increase in the expression of OsAPX5, and a slight decrease in the expression of OsAPX6, the product of which is localized in plastids and mitochondria (Fig. 4, Supplementary Material 3).

Reoxygenation had the same impact on all genes of the OsAPX family, inducing the activation of their expression both after 1 and after 24 hours. Statistically significant differences in mRNA accumulation between an hour and a day of reoxygenation were detected for OsAPX6, the expression of which increased after a day of reoxygenation, as well as for OsAPX7, the expression of which also increased after an hour of reoxygenation but began to decrease after a day of reoxygenation. During post-anoxia, OsAPX5 and OsAPX8 were the most activated. In rice roots, the changes in expression were more pronounced (see Fig. 4, Supplementary Material 3). Due to anoxia action, the expression of peroxisomal forms (OsAPX3 and OsAPX4), the plastid-mitochondrial form OsAPX5, and the stromal form OsAPX7 increased. After an hour of reoxygenation, the expression of all genes, except for OsAPX6, increased; however, after a day of reoxygenation, the mRNA levels of all genes returned to their original values. The most active genes during reoxygenation in the roots were OsAPX2, OsAPX3, and OsAPX5. In both organs, the OsAPX5 gene was the most responsive to anoxia and reoxygenation.

The family of genes encoding MDAR in rice consists of five genes. The products of the OsMDAR1 and OsMDAR2 genes are localized in peroxisomes, of OsMDAR3 and OsMDAR4, in the cytosol, and of OsMDAR5, in plastids and mitochondria (see Supplementary Material 2). In the shoots, 24 hours of anoxia led to an increase in the expression of OsMDAR2 and a decrease in the expression of OsMDAR3 (see Fig. 4, Supplementary Material 4). After an hour of reoxygenation, the expression of OsMDAR2 and OsMDAR4 peaked, and after a day of reoxygenation, there was a tendency to return to the original level of expression. The accumulation of mRNA of OsMDAR3 and OsMDAR5 also increased after an hour of reoxygenation and continued to grow after 24 hours of reoxygenation. In the roots, anoxia activated the expression of OsMDAR1, OsMDAR2, and OsMDAR4. After an hour of reoxygenation, the quantity of OsMDAR1 transcripts returned to the control normoxic level, while the expression of the OsMDAR2 and OsMDAR4 genes remained at an elevated anoxic level. After a day of reoxygenation, activity of all genes returned to the original level, and in the case of the OsMDAR1 gene, it even dropped below it.

Between the two genes encoding DHAR in rice, changes were observed in the case of OsDHAR1. In both shoots and roots, expression followed a similar pattern: 24 hours of anoxia led to a twofold decrease in transcript levels; after an hour of reoxygenation, a tendency to return to control values emerged, which were successfully restored after a day of reoxygenation (see Fig. 4, Supplementary Material 5).

Three GR genes were identified in the rice genome: OsGR1 and OsGR3 encode isoforms of the enzyme with plastidmitochondrial localization, while OsGR2 encodes a cytosolic isoform (see Supplementary Material 2). The expression of OsGR1 decreased, OsGR2 remained unchanged, and OsGR3 increased in the shoots after 24 hours of anoxia (see Fig. 4, Supplementary Material 6). After an hour of reoxygenation, the accumulation of OsGR1 mRNA returned to the normoxic level, OsGR2 increased, and OsGR3 continued to rise, exceeding the control level by 30 times. After 24 hours of reoxygenation, the amount of OsGR1 transcripts continued to increase, OsGR2 remained at the same level, and OsGR3 returned to the initial value. In the roots of rice, the changes in OsGR2 and OsGR3 were perfectly synchronized: an increase after anoxia, maintenance of the same level after an hour of reoxygenation, and restoration of the initial level after a day of reoxygenation. The expression of OsGR1 remained unchanged. OsGR3 was predominant in response to both anoxia and reoxygenation in both organs.

## Discussion

The obtained results demonstrated that under normoxic conditions, the hypoxia-sensitive plant (wheat) accumulated higher levels of ascorbic acid, while the hypoxia-tolerant plant (rice) produced more glutathione (see Fig. 1, 2). After 24 hours of anoxia and short-term (15 minutes to 1 hour) reoxygenation, there was an accumulation of oxidized forms of ascorbate and glutathione in wheat tissues. The ratio of reduced forms to oxidized forms (AsA/DHA, GSH/GSSG) decreased below one, indicating the development of oxidative stress. It is important to note that in the roots of wheat, by 24 hours of post-anoxia, the levels of the reduced antioxidants (AsA, GSH) returned to control levels (see Fig. 1, c, Fig. 2, c), whereas in the shoots, this did not occur. In wheat shoots, more DHA accumulated (see Fig. 1, a), and the total ascorbate level (AsA + DHA) decreased, indicating a greater development of oxidative processes. This is consistent with previously obtained data on higher oxidative damage to lipids in the shoots compared to the roots of wheat during anoxia and reoxygenation (Chirkova et al., 1998; Shikov et al., 2022)

In rice seedlings, the reduced forms of AGC antioxidants predominated both under normoxic conditions and during stress (see Fig. 1, b, d, Fig. 2, b, d ). This suggests an effective antioxidant defence that mitigates the development of oxidative stress. Less oxidative damage to lipids and proteins (Chirkova et al., 1998; Shikov et al., 2022) and lower hydrogen peroxide (H2O2) production (Yemelyanov et al., 2022) were observed early in rice seedlings compared to wheat.

In studies performed by other authors, the accumulation of AsA and DHA, as well as a decrease in the levels of both forms of glutathione, were demonstrated in wheat under root anoxia (Biemelt et al., 1998). A short-term reoxygenation after anoxia caused a downregulation of the level of AsA, accumulation of DHA, and GSH. Though the total content remained unchanged, anoxia led to the depletion of the AsA pool and the accumulation of DHA, while reoxygenation stimulated the restoration of DHA in barley leaves (Hordeum vulgare) (Skutnik, Rychter, 2009), and in a suspension cell culture of Arabidopsis thaliana (Paradiso et al., 2016). During prolonged anoxia, the content of all forms of low-molecular-weight antioxidants of the AGC decreased in the roots of wheat and rice seedlings, as well as in the rhizomes of the Iris species, differing in resistance to hypoxia. However, in plants resistant to hypoxia, the depletion was less pronounced (Blokhina et al., 2000). The leaves of the moderately flood-tolerant citrumelo variety CPB4475 contained high levels of AsA and GSH during prolonged waterlogging and subsequent reoxygenation (Hossain et al., 2009). Transgenic A. thaliana plants, tolerant to flooding and reoxygenation and overexpressing the MYC2 gene, which encodes a transcription factor involved in jasmonic acid signalling, accumulated more AsA and GSH during hypoxia and reoxygenation compared to wild-type and myc2 knockout mutants (Yuan et al., 2017). Therefore, it can be concluded that flood-tolerant plants are characterized by the prevalence of reduced forms of ascorbate and glutathione during oxygen deprivation and subsequent reoxygenation, which may result from their active reduction during the AGC operation.

The activity of AGC enzymes in the seedlings of the nontolerant plant (wheat) under anoxia and reoxygenation either remained unchanged or was suppressed (APX and DHAR in the shoots, MDAR and GR in the roots) (see Fig. 3). As a result, the effective functioning of the AGC became impossible. The decrease in the activity of enzymes in the ascorbate part of the AGC (APX and DHAR) may be associated with the inability of wheat shoots to restore the pre-stress levels of AsA and DHA during oxygen deficiency and subsequent reoxygenation (see Fig. 1, a). At the same time, maintenance of the activity of these enzymes may contribute to achieving the pre-stress levels in the roots of wheat (see Fig. 1, c). In the roots of the tolerant plant (rice), the activity of AGC enzymes under oxygen deficiency and post-anoxia also remained unchanged (except for MDAR at 24 hours of reoxygenation, see Fig. 3). In rice shoots, anoxia led to a decrease in the activity of APX, MDAR, and DHAR, and the reintroduction of oxygen stimulated all the enzymes of the cycle, resulting in the accumulation of reduced forms of AsA and GSH (see Fig. 1, 2).

Earlier studies have demonstrated that the deficiency or absence of O2 resulted in a decrease in the activity of all AGC enzymes in wheat roots (Biemelt et al., 1998) and in the hypoxia-grown seedlings of rice and lotus (Ushimaru et al., 1992, 2001). There is also data on some of the enzymes of the AGC for several flood-intolerant species. Thus, oxygen deficiency suppressed the activity of APX, DHAR, and GR in the leaves of cotton (Wang et al., 2019) and barley (Skutnik, Rychter, 2009), APX and GR in Welsh onion roots (Yiu et al., 2009), and APX activity in soybean (Kausar et al., 2012). O2 deficiency did not affect the activity of AGC enzymes in the roots of lupine (Garnczarska, 2005), while in the leaves of the moderately flood-tolerant citrus variety citrumelo CPB4475, it stimulated the activity of most AGC enzymes, except MDAR (Hossain et al., 2009). In the suspension cell culture of A. thaliana, the activity of APX and GR was suppressed by anoxia and stimulated by reoxygenation, while the activities of MDAR and DHAR remained unchanged (Paradiso et al., 2016). GR was stimulated during post-hypoxia, while no stimulation of AGC enzymes was observed during post-anoxia in wheat roots (Biemelt et al., 1998). Reoxygenation stimu-lated the activity of all AGC enzymes in rice and lotus seedlings (Ushimaru et al., 1992, 2001), in lupine roots (Garnczarska, 2005), and GR in barley roots (Skutnik, Rychter, 2009). The activity of GR in barley shoots, as well as the activities of APX and DHAR in the whole plant, remained unchanged during post-anoxia (Skutnik, Rychter, 2009). A comparison of plants varying in flood tolerance revealed a higher activation of APX and GR during hypoxia and reoxygenation in the tolerant forms of pigeon pea, mung bean (Sairam et al., 2009, 2011), rice varieties (Damanik et al., 2010), and ryegrass (Liu, Jiang, 2015). It can be concluded that oxygen deficiency leads to the preservation or activation of AGC enzymes in flood-tolerant plants, and the reintroduction of O2 into the environment stimulates their activity. This enables the reduction of oxidized forms of low-molecular-weight antioxidants of the cycle and ensures their efficient recyclization

For a more in-depth analysis of the AGC operating in the anoxia-tolerant rice seedlings, we have investigated the expression of genes encoding the enzymes of the cycle. Although the activity of APX in rice shoots decreased during anoxia (see Fig. 3, a), the downregulation of expression was observed only in OsAPX6, encoding the plastid-mitochondrial isoform (see Fig. 4, Supplementary Material 3). Reversely, the activation of expression was observed for OsAPX5, encoding an enzyme of the same localization, and for peroxisomal OsAPX3 in rice shoots in an anaerobic environment. The reoxygenation stimulated the accumulation of mRNA for all OsAPX, including the cytosolic OsAPX1 and OsAPX2, which corresponded to the increase in APX activity to the control normoxic levels. The post-anoxic conditions tended to activate predominantly the OsAPX5 and OsAPX8 genes, which encode plastid isoforms. In the roots, enzyme activity remained unchanged, but the expression levels of peroxisomal forms (OsAPX3 and OsAPX4) and plastid forms (OsAPX5 and OsAPX7) increased already under anoxia, with the addition of the cytosolic (OsAPX1 and OsAPX2) and the plastid form (OsAPX8) during post-anoxia. The greatest activation was observed for OsAPX2, OsAPX3, and OsAPX5, corresponding to the cytosolic, peroxisomal, and plastid-mitochondrial forms, respectively. The OsAPX5 was activated more strongly than other genes and in both organs of the seedling.

There is limited information on the expression of APX genes in the literature. In soybean seedlings, flooding suppressed the expression of cytosolic APX genes, GmAPX1 and GmAPX2 (Nishizawa et al., 2013). In pigeon pea and mung bean, the hypoxia-tolerant forms were characterized by increased expression of genes encoding cytosolic APX under hypoxia, compared to the forms that are intolerant to flooding (Sairam et al., 2009, 2011). An increased expression of OsAPX1 during flooding was detected in a submergencetolerant rice variety carrying the Sub1A allele (Parlanti et al., 2011). The flood-tolerant transgenic line of A. thaliana, expressing the MYC2 gene, and the wild-type plants were characterized by the flooding-induced activation of genes en- coding AGC enzymes (AtAPX2, AtMDHAR3, AtDHAR1, and AtGR1) (Yuan et al., 2017). The mRNA level of AtAPX2, encoding the cytosolic isoform, also increased during anoxia in suspension cell culture of Arabidopsis, but it rose even more during a short-term reoxygenation, similar to other cytosolic forms (AtAPX1 and AtAPX6). The other APXs genes were not investigated (Paradiso et al., 2016). We demonstrated the activation of APX genes encoding cytosolic forms in rice only under post-anoxia (see Fig. 4, Supplementary Material 3).

In our experiments, the activity of intermediate reductases of the AGC (MDAR and DHAR) changed similarly in the shoots: it decreased during anoxia and increased above the control level during reoxygenation. In the case of DHAR, this pattern coincided with changes in the expression of OsDHAR1 (see Fig. 4, Supplementary Material 5). The decrease in MDAR activity in the shoots occurred alongside a decline in transcripts of OsMDAR3, encoding the cytosolic form, and the accumulation of transcripts of the peroxisomal form OsMDAR2 (see Fig. 4, Supplementary Material 4). The expression of OsMDAR2 and OsMDAR4, encoding the cytosolic isoforms, increased during a short-term reoxygenation. After 24 hours of post-anoxia, the expression of OsMDAR3 and OsMDAR5, encoding the cytosolic and plastid-mitochondrial forms respectively, increased as well. In the roots, despite the absence of changes in the enzyme activity, anoxia activated the expression of OsMDAR1, OsMDAR2, and OsMDAR4. As in the shoots during reoxygenation, the quantity of mRNA of OsMDAR2 and OsMDAR4 increased. In the scientific literature, only the aforementioned article by L.-B. Yuan and coauthors (2017) was found, which investigated the expression of genes encoding AGC reductases during hypoxia and posthypoxia. The maintenance of the GR activity during anoxia in both shoots and roots of rice, as well as the activation during reoxygenation in the shoots, is primarily associated with the expression of OsGR2 and OsGR3, encoding the cytosolic and plastid-mitochondrial isoforms, respectively (see Fig. 4, Supplementary Material 6).

Therefore, in most cases, oxygen deficiency induced the activity of the genes encoding the peroxisomal, plastid, and mitochondrial forms of AGC enzymes, and upon returning to the normoxic conditions, there was also an enhancement in the expression of the genes encoding the cytoplasmic forms. The involvement of different enzyme isoforms may be an important physiological mechanism for cell adaptation during the transition from oxygen-deficient conditions to reoxygenation. On the other hand, the addition of the cytoplasmic forms of enzymes to the organellar ones, which were already activated during anoxia, might be a consequence of increased oxidative stress upon the return of the oxygen levels to normoxic values during reoxygenation. In most cases, the activation of AGC enzymes during post-anoxia corresponded to the changes in their gene expression.

## Conclusion

The comprehensive study of the AGC during anoxia and subsequent reoxygenation demonstrated the inactivation of APX and DHAR in the shoots, as well as MDAR and GR in the roots of the intolerant plant (wheat). This disruption compromised the efficient functioning of the cycle, leading to the accumulation of oxidized forms of ascorbate and glutathione and contributing to the development of significant oxidative damage. In the flood-tolerant plant (rice), the enzyme activity of the AGC was preserved under oxygen deficiency, and reoxygenation stimulated it, including the transcription level. This type of response possibly enables the recyclization of low-molecular-weight antioxidants in the cycle, providing antioxidant protection and preventing the development of oxidative stress. Thus, the resistance of plants to oxygen deficiency includes the resistance mechanisms to oxidative stress.

## Conflict of interest

The authors declare no conflict of interest.
